# The Digestive Vacuole of the Malaria Parasite: A Specialized Lysosome

**DOI:** 10.3390/pathogens13030182

**Published:** 2024-02-20

**Authors:** Mark F. Wiser

**Affiliations:** Department of Tropical Medicine and Infectious Disease, Tulane University School of Public Health and Tropical Medicine, New Orleans, LA 70112-2824, USA; wiser@tulane.edu; Tel.: +1-504-988-2507

**Keywords:** *Plasmodium*, malaria, digestive vacuole, food vacuole, endocytosis, lysosome, endosome, hemozoin, hematozoans, anti-malarial

## Abstract

The malaria parasite resides within erythrocytes during one stage of its life cycle. During this intraerythrocytic period, the parasite ingests the erythrocyte cytoplasm and digests approximately two-thirds of the host cell hemoglobin. This digestion occurs within a lysosome-like organelle called the digestive vacuole. Several proteases are localized to the digestive vacuole and these proteases sequentially breakdown hemoglobin into small peptides, dipeptides, and amino acids. The peptides are exported into the host cytoplasm via the chloroquine-resistance transporter and an amino acid transporter has also been identified on the digestive vacuole membrane. The environment of the digestive vacuole also provides appropriate conditions for the biocrystallization of toxic heme into non-toxic hemozoin by a poorly understood process. Hemozoin formation is an attribute of *Plasmodium* and *Haemoproteus* and is not exhibited by other intraerythrocytic protozoan parasites. The efficient degradation of hemoglobin and detoxification of heme likely plays a major role in the high level of replication exhibited by malaria parasites within erythrocytes. Unique features of the digestive vacuole and the critical importance of nutrient acquisition provide therapeutic targets for the treatment of malaria.

## 1. Introduction

Malaria is a common human disease in the tropics that exhibits substantial morbidity and mortality. The causative agent of malaria is a protozoan pathogen in the genus *Plasmodium*. *Plasmodium* and related Haemosporida (Apicomplexa) are dixenic parasites that infect vertebrate hosts, which include reptiles, birds, and mammals, and are transmitted by dipteran vectors [1]. A major characteristic of haemosporidians is an intraerythrocytic stage during the infection of the vertebrate host which is characterized by substantial proliferation. Residence within erythrocytes has certain advantages such as immune system avoidance and facilitation of vector transmission via blood-feeding arthropods. However, the erythrocyte may not be very accommodating from a nutritional perspective due to its rather simple composition and relatively low metabolism. The erythrocyte is approximately 95% hemoglobin and does not express the full gambit of metabolic pathways found in most cells. This raises questions about how intraerythrocytic parasites can exploit their host cell to obtain sufficient metabolites for survival and reproduction. And in some cases, this reproduction is quite notable with 30–40 progeny being produced in the order of days. A major source of nutrition for the intraerythrocytic parasite is the digestion of host cell hemoglobin as described herein.

## 2. Life Cycle

The physiology of intraerythrocytic parasitism has been best studied in mammalian malaria parasites, and especially in *P. falciparum* due to its importance as a human pathogen and the ability to culture this parasite in vitro. The malaria parasite exhibits a complex life cycle involving mosquito transmission and a transient infection of the liver before infecting erythrocytes [2]. Infection of erythrocytes is initiated by merozoites which are initially released from infected liver cells. After invading erythrocytes, parasites undergo a trophic period characterized by parasite growth. The early trophozoite stage is often called the ring stage which lasts approximately half of the erythrocytic stage replicative cycle. The late trophozoites continue to increase in size and subsequently develop into stages called schizonts. The beginning of schizogony is marked by nuclear replication without cytoplasmic division resulting in the formation of multinucleated schizonts. Schizonts divide by a segmentation process to produce numerous merozoites that are released by rupture of the infected erythrocyte. These newly released merozoites reinitiate the intraerythrocytic replicative cycle after invading new erythrocytes to produce a chronic infection that often lasts for months. The repeated rounds of blood-stage schizogony are responsible for the clinical manifestations and pathology of the disease. Some of the merozoites, instead of undergoing asexual replication, develop into sexual forms called gametocytes. Mature gametocytes are uninucleated parasites that are infective to mosquitoes and play a key role in transmission.

During the intraerythrocytic stage, the parasite ingests the host cell cytoplasm and essentially converts the mass of the erythrocyte into its own mass. Thus, mature schizonts and mature gametocytes nearly fill the entire erythrocyte cytoplasm. During these maturation processes, approximately 70% of the soluble content of the infected host erythrocyte is ingested by the parasite [3]. Ingestion of the erythrocyte cytoplasm, which is primarily hemoglobin, is mediated by endocytosis and the endocytosed material forms the digestive vacuole. Within the digestive vacuole, also called the food vacuole, hemoglobin is broken down into amino acids which can then be utilized by the parasite [4,5]. The parasite also induces nutrient channels on the infected erythrocyte for the acquisition of metabolites [6,7].

## 3. Endocytosis and the Digestive Vacuole

Endocytosis is a general term referring to the uptake of substances that involves surrounding the material to be taken up with the plasma membrane and the enclosure of that material into membrane-bound vesicles. The engulfment of large particulate matter is called phagocytosis, and pinocytosis has historically been used to describe the engulfment of fluid. There are distinct types of fluid-phase endocytosis based on the size and volume of material being taken up and the mechanisms involved in the formation of endocytic vesicles [8,9]. For example, a major form of endocytosis is clathrin-mediated endocytosis which has historically been called receptor-mediated endocytosis [10]. Clathrin-independent endocytosis is poorly defined in regards to molecular components and their nomenclature is imprecise with various names [11]. Endocytic vesicles can fuse to form the endosome, or the endocytic vesicles can fuse with a pre-existing endosome [12,13]. Endosomes direct their content to other subcellular compartments, such as lysosomes. Alternatively, lysosomes can fuse with endosomes to form the lysosomal compartment.

**Figure 1:**
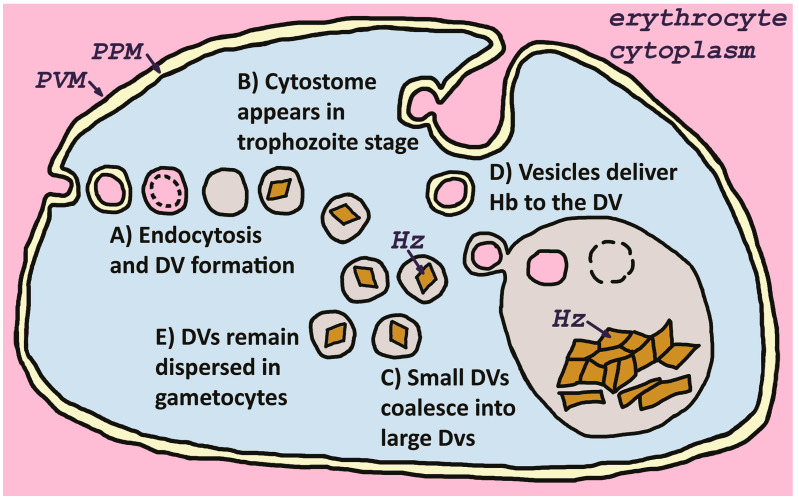
Endocytosis and digestive vacuole formation. Host cell cytoplasm is taken up by fluid-phase endocytosis involving the parasite plasma membrane (PPM) and the parasitophorous vacuolar membrane (PVM) leading to the formation of double membrane vesicles (A). The inner membrane, corresponding to the PVM, is rapidly degraded and the vesicles form small digestive vacuoles (DV) in which hemoglobin is degraded and hemozoin (Hz) forms. As the parasite matures, cytostomes appear and these serve as a major site for endocytosis (B). Also, as the parasite matures the small dispersed digestive vacuoles coalesce into large digestive vacuoles (C). Thereafter, hemoglobin (Hb)-containing vesicles deliver their content to the large digestive vacuoles (D). In gametocyte stages the digestive vacuoles do not coalesce and remain dispersed in the parasite cytoplasm (E).

The endocytic uptake of host erythrocyte cytoplasm was described in the early days of electron microscopy of the malaria parasite, and it was correctly surmised that this endocytosis was related to the digestion of hemoglobin [14]. Endocytosis producing small vesicles commences during the ring stage shortly after the parasite invades the host erythrocyte [15]. The intraerythrocytic parasite is surrounded by a membrane partially of host origin [16,17], called the parasitophorous vacuolar membrane (PVM). Therefore, endocytosis results in double-membrane vesicles with the inner membrane originating from the PVM [18]. The inner membrane rapidly disappears and initially the vesicles function as individual digestive vacuoles [19]. As the parasite grows and develops, the small independent digestive vacuoles coalesce into larger digestive vacuoles (Figure 1). The timing of this coalescence varies according to species with some species forming a large digestive vacuole during the early trophozoite stage, whereas in other species the large digestive vacuole appears in the late trophozoite stage [4]. In addition, it has long been recognized that the small digestive vacuoles do not coalesce into larger digestive vacuoles in the gametocytes, but remain dispersed as small vesicles [20].

Coincident with the maturation of trophozoites is the formation of cytostomes [21]. Cytostomes are tube-like openings on the parasite surface that serve as the focal points of endocytosis. On average, two and a half cytostomes are found per parasite [22]. Double-membrane vesicles are pinched from the base of the cytostome and these vesicles fuse with the digestive vacuole releasing the inner vesicle formed from the PVM. This inner membrane is rapidly degraded to release the hemoglobin. Other membrane invaginations have been described on the parasite surface and there may be multiple mechanisms of host cytosol uptake [23].

### 3.1. Molecular Components of Endocytosis

The endosomal pathway of the malaria parasite has not yet been extensively characterized at the molecular level and only a few potential endocytosis-associated proteins have been identified [5]. Through inhibitor and genetic studies, several *Plasmodium* orthologs of proteins known to be involved in endocytosis or trafficking to the lysosome have been implicated (Table 1). Endocytosis in *Plasmodium* appears to be clathrin independent, even though the clathrin adaptor protein complex (AP-2) appears to be involved [24,25]. In most eukaryotes, the AP-2 complex in conjunction with clathrin forms a major fluid-phase endocytic pathway [26]. Thus, the AP-2 complex may have a role in *Plasmodium* that is distinct from other eukaryotes. For example, *Toxoplasma* clathrin is not found on the plasma membrane and is associated with post-Golgi trafficking [27].

The *Plasmodium* AP-2μ subunit interacts with Kelch13, which has been localized to the neck region of the cytostome [25]. Other proteins that interact with Kelch13 include the de-ubiquitinase UBP1 and the *Plasmodium* homolog of the endocytosis protein Eps15. *P. falciparum* Eps15 has been demonstrated to be involved in endocytosis and possibly lipid storage [36]. Presumably, this Kelch13-Eps15-AP-2m-UBP1 protein complex mediates the uptake of the host cell cytoplasm at the cytostome.

### 3.2. Digestive Vacuole Properties and Formation

The *Plasmodium* digestive vacuole exhibits similarities to late endosomal compartments in that it is acidic (pH 5.0–5.4) and contains hydrolytic enzymes. However, the digestive vacuole is lacking in most nonproteolytic hydrolases suggesting a specialization in the degradation of hemoglobin [37]. The acidic pH of the *Plasmodium* digestive vacuole is maintained by two proton pumps. As is also seen in plants, one pump is a V-type H^+^-ATPase and the other pump is a H^+^-pyrophosphatase [38]. Proteomic analysis of the digestive vacuole has identified 116 proteins [39]. Many of these are the previously characterized proteases of the digestive vacuole as well as proteins potentially involved in trafficking and biogenesis of the digestive vacuole. The previously characterized proteases include aspartic proteases (called plasmepsins), cysteine proteases (called falcipains), metalloproteases (called falcilysins), and exopeptidases (Table 2).

The endocytic pathway in *Plasmodium* does not appear to involve the fusion of preformed lysosomes with the endosomes, as is typical of many eukaryotes. In addition, little is known about the targeting of hydrolytic enzymes to the digestive vacuole. Plasmepsin-2 is transported through the secretory pathway and taken up by endocytosis along with the hemoglobin substrate [49]. Dipeptidyl aminopeptidase-1 may be trafficked to the digestive vacuole via the parasitophorous vacuole [46]. Thus, digestive vacuole hydrolases may be first secreted into the parasitophorous vacuole or host erythrocyte cytoplasm and then subsequently endocytosed. Falcipains, on the other hand, are first trafficked to the parasite plasma membrane en route to the digestive vacuole [50]. Falcipains also activate the plasmepsins [51]. It is not clear how the proton pumps are targeted to the digestive vacuole, but presumably they are trafficked to specific sites on the parasite plasma membrane. The sites of endocytosis on the parasite plasma membrane, including the cytostome, may possibly be enriched for the proton pumps and some of the hydrolytic enzymes. This implies that digestive vacuoles are generated de novo as part of endocytosis, which is consistent with the endocytic vesicles directly functioning as individual digestive vacuoles [19].

## 4. Hemoglobin Catabolism

**Figure 2:**
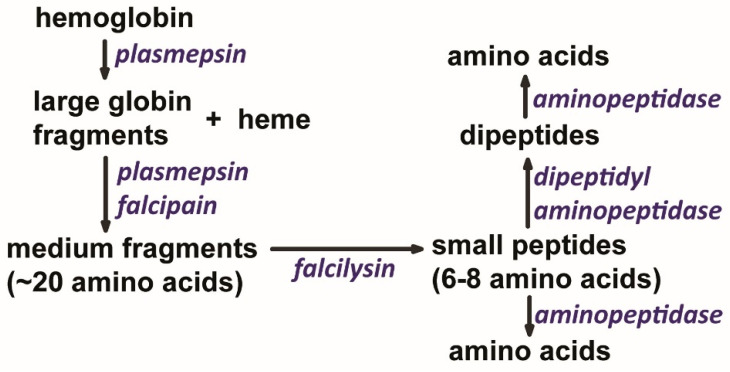
Ordered degradation of hemoglobin within the digestive vacuole. Initially, the combined actions of plasmepsins and falcipains break globin into medium-sized fragments that are subsequently broken down into small fragments by falcilysin. Following degradation by these endopeptidases, some of the small peptides are converted to dipeptides and amino acids by exopeptidases. Thus, the end-products of hemoglobin digestion are small peptides, dipeptides, and amino acids which are translocated to the parasite cytoplasm.

The *Plasmodium* digestive vacuole contains numerous endo- and exo-proteases, and hemoglobin is broken down by the sequential action of these proteases in an ordered process [52]. In addition, gene knockout studies have demonstrated that no single food vacuole protease is essential, indicating functional redundancy [53]. The various endo-proteases participate in an ordered digestion of hemoglobin into large peptides, medium peptides, and small peptides (Figure 2). The initial cleavage likely occurs between residues 33 (phenylalanine) and 34 (leucine) of the α-subunit by a plasmepsin [54]. This proteolytic site is called the hinge region and is a highly conserved domain responsible for holding the hemoglobin tetramer together. Cleavage at this site likely results in an unraveling of the hemoglobin molecule and the exposure of additional proteolytic sites which leads to further degradation to medium-sized peptides by the plasmepsins and falcipains. Degradation of medium-sized polypeptides is mediated by falcilysins, and small polypeptides are degraded into dipeptides and amino acids by exopeptidases in digestive vacuole (Table 2). The final products of these various proteases and peptidases are small peptides consisting of 5–10 amino acids, dipeptides, and amino acids that are transported to the cytoplasm of the parasite. Neutral aminopeptidases in parasite cytoplasm convert the peptides and dipeptides into amino acids [55].

It is generally presumed that hemoglobin is digested to supply amino acids for the synthesis of parasite proteins. However, less than one-fifth of the amino acids obtained from the digestion of hemoglobin are incorporated into parasite proteins [56], and large amounts of amino acids are effluxed into the host erythrocyte [57]. Possible explanations for this discrepancy between the amount of hemoglobin ingested and amino acid utilization might be explained as a means to meet the space requirements of the growing parasite [58]. In addition, digestion of hemoglobin may help balance the intracellular osmotic pressure and thereby prevent premature lysis of the host erythrocyte [59]. Presumably, amino acids could also serve as an energy source through glucogenesis.

### 4.1. Digestive Vacuole Membrane Transporters

Amino acids and peptides produced by the catabolism of hemoglobin are transported from the digestive vacuole to the parasite cytoplasm. The chloroquine resistant transporter (CRT) likely plays a major role in this transport, in addition to its role in drug resistance and chloroquine efflux [60]. CRT is a member of the drug/metabolite transporter superfamily [61] and was originally identified via a cross between drug-resistant and drug-sensitive parasites [62]. The natural function of CRT is likely the transport of peptides consisting of 4–11 amino acids from digestive vacuole to parasite cytoplasm [63]. CRT may be a proton symporter that utilizes the proton-motive force of the digestive vacuole to move solutes across the membrane [64].

A predicted amino acid transporter, called PfAAT1, has also been localized to the *P. falciparum* digestive vacuole [65]. Its role in amino acid transport has not yet been demonstrated, but a mutation in PfAAT1 S258L does potentiate chloroquine resistance [66]. Initially, there was a lot of focus on a member of the ATP-binding cassette (ABC) superfamily of transporters, known as either P-glycoprotein homolog-1 or multiple drug resistance-1 (MDR-1), as the peptide/amino acid transporter. However, this ABC transporter is now known to import material, including possibly drugs, from the cytoplasm into the digestive vacuole [67].

### 4.2. Heme Detoxification

The proteolysis of hemoglobin results in the release of heme and this release of heme is associated with the oxidation of iron from the ferrous state (Fe^2+^) to the ferric state (Fe^3+^). Ferric iron is a pro-oxidant and catalyzes the production of reactive oxygen species (ROS), such as superoxide and hydrogen peroxide [68]. Ferryl iron (Fe^4+^) has also been suggested to be involved in the production of ROS [69]. These ROS increase oxidative stress including the alkylation of proteins and the peroxidation of lipids. In addition, hematin has detergent-like properties, and combined with lipid peroxidation, can lyse biological membranes [70]. Since the concentration of hemoglobin is in the millimolar range and approximately 70% of the hemoglobin is catabolized, high levels of toxic heme molecules are released during the digestion of hemoglobin. Heme is detoxified through a poorly understood biocrystallization process that results in the formation of insoluble hemozoin which is no longer toxic [71,72].

Hemoglobin crystals appear in the trophozoite stage initially in the small individual digestive vacuoles and then later as large hemozoin crystals after the coalescence of the digestive vacuoles. Hemozoin crystals are also called the malarial pigment and this pigment was instrumental in the discovery of the malaria parasite [73]. During schizogony and merozoite formation, the hemozoin-containing digestive vacuole is segregated into a membrane-bound residual body that is released with the rupture of the infected erythrocyte. Released hemozoin may contribute to severe disease [74,75] and the inflammatory response associated with malaria [76].

### 4.3. Hemozoin Formation

Hematin forms dimers known as β-hematin, which is chemically equivalent to hemozoin [77]. It has been suggested that biocrystallization could occur spontaneously under conditions of low pH and high hematin concentration [78]. However, in addition to the acidic environment of the digestive vacuole, it appears that lipids and the digestive vacuole membrane are crucial for the formation of hemozoin crystals [79,80,81,82,83]. It has been suggested that the digestive vacuole membrane may serve as an interface for the nucleation of hemozoin crystals [72].

The exact mechanism of hemozoin formation is unknown and proteins may also be involved. A *Plasmodium* lipocalin-like protein has been implicated in the function of the digestive vacuole [84,85]. Lipocalins bind to small hydrophobic molecules and are often involved in lipid transport or oxidative stress responses. *Plasmodium* lipocalin is localized to both the parasitophorous vacuole and the digestive vacuole. Modulation of *Plasmodium* lipocalin levels suppress hemozoin formation [85] and increase susceptibility to ROS [84]. Parasite proteins previously implicated in hemozoin formation include histidine-rich proteins [86] and heme detoxification protein [87]. However, histidine-rich proteins are primarily localized to the host erythrocyte cytoplasm and parasites deficient in histidine-rich proteins still produce hemozoin. Similarly, heme detoxification protein is localized to the parasite cytoplasm and denaturing this protein does not abrogate hemozoin formation. Therefore, the role of histidine-rich protein and heme detoxification protein in hemozoin formation is questionable.

## 5. Anti-Malarials and the Digestive Vacuole

Many highly efficacious anti-malarial drugs target the digestive vacuole [5,88], and especially notable are 4-aminoquinolines, such as chloroquine, and artemisinin derivatives. Chloroquine has long been known to accumulate to high levels in the digestive vacuole. This accumulation may be due in part to the weak base properties of chloroquine and its subsequent protonation in the acidic digestive vacuole. The accumulation of chloroquine in the digestive vacuole may also be due to its binding to hemozoin [89,90]. Regardless of the mechanism(s), chloroquine is concentrated to millimolar levels within the digestive vacuole [91] and interferes with hemozoin formation [92,93]. Specifically, chloroquine may bind to the hemozoin crystal and thereby prevent the further addition of β-hematin dimers to the growing crystal [94]. This drug-mediated failure to detoxify heme increases oxidative stress, lyses membranes, and leads to parasite death.

Artemisinins are potent prodrugs that require activation [95]. The first step in this activation is the rapid and non-specific conversion of artemisinin derivatives to dihydroartemisinin. The second step is parasite specific and requires hemoglobin uptake and digestion [96]. Free heme in the digestive vacuole interacts with artemisinin and this interaction generates free radicals [97]. The activated artemisinin alkylates heme and prevents it from forming hemozoin crystals [98]. As with the 4-aminoquinolines, artemisinin derivatives also block heme detoxification and increase oxidative stress in the parasite resulting in parasite death. Heme-mediated activation of artemisinin also results in widespread parasite protein alkylation [99]. This would contribute to parasite death by inhibiting key proteins in essential parasite pathways.

## 6. Other Intraerythrocytic Protozoan Parasites

**Figure 3:**
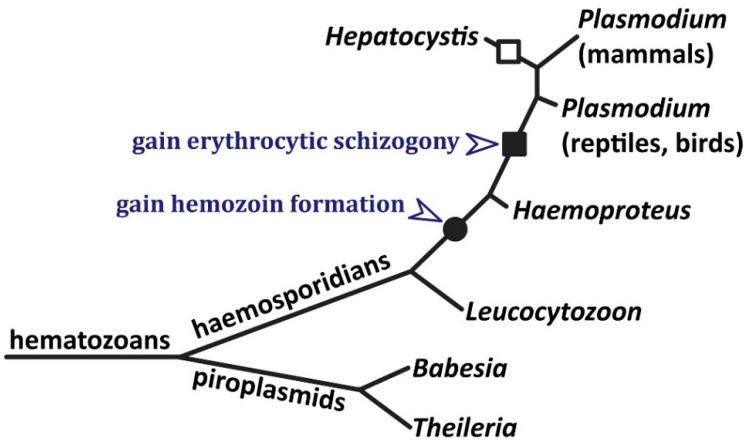
Phylogenetic relationships among the hematozoans and the gain of hemozoin formation. Hematozoans are characterized by one stage of the life cycle involving the infection of erythrocytes. The phylogenetic tree shows probable branching order of major genera [101,103]. Branch lengths do not depict evolutionary distances and branches do not depict complexity of the genera. *Hepatocystis* may branch within the mammalian *Plasmodium* clade [1]. The filled circle shows the gain of hemozoin formation, and the filled square shows the gain of schizogony during the erythrocytic stage. Erythrocytic schizogony is lost in *Hepatocystis* (open square).

Intraerythrocytic protozoa are only found in the Apicomplexa [100]. The four groups of apicomplexans exhibiting intraerythrocytic stages are haemosporidians, piroplasmids, haemogregarines, and haemococcidians. Haemosporidians and piroplasmids form a group called hematozoa and haemogregarines and haemococcidia are within the coccidian clade—a sister group of the hematozoans. By far, most of our knowledge on feeding mechanisms within the infected erythrocyte is from mammalian malaria parasites which are members of the haemosporidian clade. Several haemosporidians produce hemozoin and the presence of hemozoin is used as a taxonomic tool within this group [101]. The ability to form hemozoin emerged only once and is found in the *Plasmodium* and *Haemoproteus* genera (Figure 3). This emergence of hemozoin formation may have occurred between 57 and 69 million years ago [102]. There are no reported losses of hemozoin production within this clade indicating it is a robust biological advantage. Hemozoin formation is not found in the piroplasmids, haemogregarines, or haemococcidians, suggesting that these species do not extensively digest hemoglobin or have other mechanisms to detoxify heme. This gain of hemozoin formation in just one clade of erythrocyte-infecting protozoa strongly suggests a genetic basis for hemozoin formation. Identification of the gene(s) involved in hemozoin formation may provide additional therapeutic targets for the treatment of malaria.

Although probably not related to hemozoin formation, schizogony during the erythrocytic stage emerged after hemozoin production and is used to distinguish *Plasmodium* from *Haemoproteus* [101]. Erythrocytic stage schizogony has been lost in *Hepatocystis*.

Due to their veterinarian significance, some work has been carried out on the piroplasmids. However, most of this work has been limited to ultrastructural studies and feeding mechanisms of the piroplasmids have not been extensively characterized [104]. It has been suggested that *Babesia* takes up hemoglobin via endocytosis [105]. However, no digestive vacuole has been described [106] and the parasite does not have cytostomes [105,106]. Cytostomes and digestive vacuoles have been reported in intraerythrocytic *Theileria* [107,108], and there may be a connecting channel between the Theileria’s cytostome and its digestive vacuole [108]. It has been suggested that feeding in *Babesia* is mediated by a special organelle composed of tightly coiled double membranes located partly inside and partly outside the parasite [109]. It is presumed that extracellular digestion of host cytoplasm takes place through this organelle, and thus, feeding relies heavily on osmotrophy. In this regard, it is speculated that *Babesia* may secrete proteases into host erythrocyte cytoplasm [110]. Other than falcipain-2, *Babesia* does not have orthologs of the hemoglobin degrading proteases of *Plasmodium* [104]. *Babesia* does not produce hemozoin [111], and expectedly, chloroquine does not work against *Babesia* nor *Theileria* [112]. A tubule between parasite and host erythrocyte membrane is reported in *Babesia* [113] and *Theileria* [114]. It is not clear if this tubule functions in direct feeding from the host plasma, or if the tubule is involved in excretion of material from the parasite and infected erythrocyte.

## 7. Summary

The malaria parasite has adapted to life within the erythrocyte by evolving an efficient mechanism to ingest the host cell hemoglobin. The first step of this hemoglobin ingestion is the endocytosis of the host erythrocyte cytoplasm. A few proteins known to be involved in endocytosis have been identified in *Plasmodium*, but the exact mechanism(s) are not known. Interestingly, clathrin does not appear to be involved in endocytosis even though the adapter protein complex does appear to be involved. One notable unique feature is the formation of double-membrane vesicles that include the parasitophorous vacuolar membrane.

**Figure 4:**
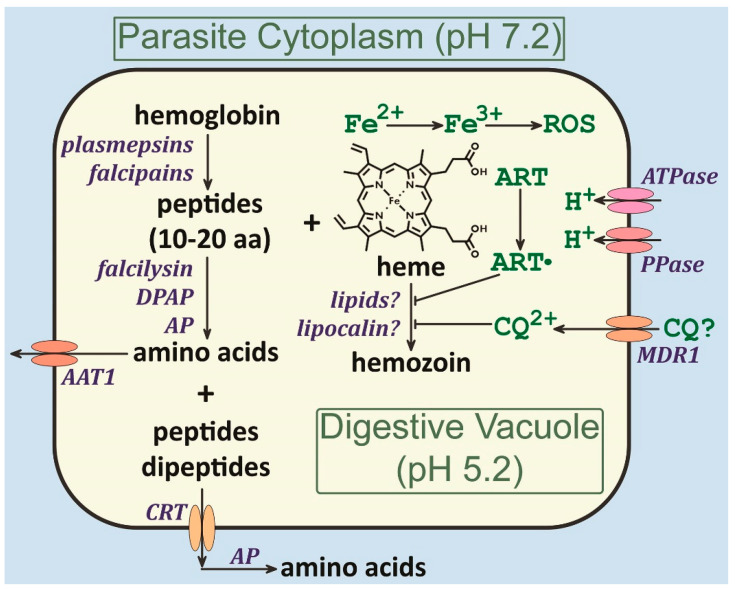
The *Plasmodium* digestive vacuole. Summary of the activities associated with the *Plasmodium* digestive vacuole as discussed in this paper. Abbreviations: AAT = amino acid transporter; AP = aminopeptidase; ART = artemisinin (dot indicates activated free radical); CQ = chloroquine; CRT = chloroquine resistance transporter; DPAP = dipeptidyl aminopeptidase; MDR = multi-drug resistance; PP = pyrophosphatase; ROS = reactive oxygen species.

A digestive vacuole is formed from the endocytic vesicles and hemoglobin is broken down into amino acids and peptides. This *Plasmodium* digestive vacuole is a specialized lysosome that efficiently degrades hemoglobin and detoxifies heme (Figure 4). Hemoglobin degradation involves the sequential action of several proteases that convert globin into peptides, dipeptides, and amino acids. These peptides and amino acids are exported into the cytoplasm of the parasite with transporters on the digestive vacuole membrane. Heme is primarily detoxified through the formation of hemozoin via a biocrystallization process. The exact mechanisms of this biocrystallization are not known, but this process evolved within the haemosporidian clade. This biocrystallization is critical for parasite survival since anti-malarial drugs that interfere with this process are highly efficacious.

## Figures and Tables

**Table 1:** Proteins implicated in malaria parasite endocytosis.

Protein	Comments
vacuolar sorting protein 45	Inactivation leads to the accumulation of vesicles and prevents the delivery of hemoglobin to the digestive vacuole [28]
Rab5a	Implicated in hemoglobin uptake and transport to digestive vacuole [23]
dynamin like protein-1	Inhibitors reduce hemoglobin uptake [29]
actin	Inhibition of actin polymerization increases hemoglobin containing vesicles [22,30]
phosphatidylinositol-3 kinase	Inhibitors block trafficking of hemoglobin to the digestive vacuole [31]
phosphoinositide-binding protein	Localized to the digestive vacuole and plays a role in trafficking of hemoglobin [32]
protein prenylation	Inhibitors cause deformation of digestive vacuole and mislocalization of Rab5 [33]
μ subunit of AP-2 adaptin complex	Implicated in clathrin-independent endocytosis [24]
Kelch13	Localized to cytostomes [25] and mutations [34] or reduced abundance [35] impairs hemoglobin catabolism
Eps15	Localized to a large multi-vesicular structure near the digestive vacuole [36]

**Table 2:** Proteases of the *Plasmodium* digestive vacuole.

Class	Protease	Comments
Aspartic Proteases [40]	Plasmepsin-1 (PM1)	PM4 is found in all *Plasmodium* species and PM1, PM2, and HAP are paralogs of PM4 and are only found in *P. falciparum* [41]
	Plasmepsin-2 (PM2)	
	Plasmepsin-4 (PM4)	
	Histoaspartic protease (HAP), aka plasmepsin-3	
Cysteine proteases [42]	Falcipain-2 [43]	Falcipain-2 and -3 exhibit 70% sequence identity and falcipain-2′ is a nearly identical copy of falcipain-2
	Falcipain-2′	
	Falcipain-3 [44]	
Metalloprotease	Falcilysin [45]	Prefers peptides < 20 amino acids long
Exopeptidases	Dipeptidyl aminopeptidase-1 [46]	Generates dipeptides from hemoglobin-derived peptides
	Aminopeptidase P [47]	Possibly functions in both the digestive vacuole and the parasite cytoplasm
	M1 alanyl aminopeptidase [48]	Inhibition caused swelling of the parasite digestive vacuole and prevented proteolysis of hemoglobin-derived peptides

## Data Availability

The original contributions presented in the study are included in the article, further inquiries can be directed to the corresponding author.
